# Phenolic Compounds Obtained from *Olea europaea* By-Products and Their Use to Improve the Quality and Shelf Life of Meat and Meat Products—A Review

**DOI:** 10.3390/antiox9111061

**Published:** 2020-10-29

**Authors:** Paulo E. S. Munekata, Gema Nieto, Mirian Pateiro, José Manuel Lorenzo

**Affiliations:** 1 Centro Tecnolóxico da Carne de Galicia, rúa Galicia n◦ 4, Parque Tecnolóxico de Galicia, San Cibrao das Viñas, 32900 Ourense, Spain; paulosichetti@ceteca.net (P.E.S.M.); mirianpateiro@ceteca.net (M.P.); 2 Department of Food Technology, Nutrition and Food Science, Veterinary Faculty University of Murcia, Campus Mare Nostrum, 30100 Espinardo, Murcia, Spain; gnieto@um.es; 3 Área de Tecnología de los Alimentos, Facultad de Ciencias de Ourense, Universidad de Vigo, 32004 Ourense, Spain

**Keywords:** oleupein, hydroxytyrosol, tyrosol, leaves, pomace, wastewater, lipid and protein oxidation, antimicrobial activity, olive tree

## Abstract

Consumers are interested in consuming clean label foods. Replacing synthetic additives with natural alternatives (especially sources rich in polyphenols) is a valid solution to produce and also preserve foods, especially meat and meat products. *Olea europaea* leaves and olive pomace and wastewater contain polyphenols that can be explored in this context. In this review, we summarize the main aspects related to the phenolic composition, extraction conditions, antimicrobial potential, and antioxidant activity (in vitro and in vivo) of *Olea europaea* leaves, olive pomace and wastewater as well as their applications in the production of meat and meat products. This review found evidence that extracts and isolated polyphenols from the *Olea europaea* tree and olive processing by-products can be explored as natural antioxidant and antimicrobial additives to improve the preservation of meat and meat products. The polyphenols found in these residues (especially oleuropein, hydroxytyrosol and tyrosol) increased the redox state in the main meat-producing animals and, consequently, the oxidative stability of fresh meat obtained from these animals. Moreover, the extracts and isolated polyphenols also improved the shelf life of fresh meat and meat products (as additive and as active component in film) by delaying the growth of microorganisms and the progression of oxidative reactions during storage. The accumulated evidence supports further investigation as a natural additive to improve the preservation of reformulated muscle products and in the production of edible and sustainable films and coatings for fresh meat and meat products.

## 1. Introduction

The cultivated olive tree (*Olea europaea*) belongs to the genus *Olea*, which is currently cultivated around the globe due to its capacity to thrive under unfavorable conditions such as semi-arid environment, limited water supply and high temperatures during summers [1]. These characteristics were of great importance for the extensive cultivation and the production of olive fruits in the Mediterranean basin, which was the region with the most products in the world [2]. In terms of the global market, the production of olive fruits is destined to be consumed in the form of table olives or is processed to obtain olive oil that accounted for more than 3284 and 3379 million tons in 2018, respectively [2,3].

The large production of olive fruits is motivated by the highly appreciated odor, flavor, and taste as well as nutritional quality associated with consumption of table olives and oil [3,4,5]. Moreover, olive oil is one of the pillars of the Mediterranean diet, which is associated with an increased healthy status, reduced risk of developing cardiovascular diseases, diabetes, weight management and other illness, and also decreased all-cause mortality [6,7].

Because of the importance of olive oil, several standard regulations have been defined by both national and international organizations, such as the European Commission standards [8], United States Department of Agriculture [9], and International Olive Council [10], that indicate the limits for fatty acids, sterols, and wax content and composition, organoleptic descriptors, absorbance in specific UV lengths, and polyphenols. Although the sector of olive oil is well regulated to reduce the occurrences of intentional adulteration for financial gains, this product ranks in the top of the most reported fraudulent products in the European Union in 2019 (in the “oils and fats” category) [11]. In order to improve the identification of frauds related to olive oil, advanced analytical techniques (such as mass, NMR and vibrational spectroscopy) with posterior data analysis applying chemometrics is a strategy of great value. The importance of this approach is due to the high-throughput, reproducibility, robustness, and higher sensitivity in relation to conventional analytical methods [12,13].

The high production, nutritional importance, health benefits, and commercial value are also followed by higher amounts of residues such as leaves, pomace, and wastewater [14,15]. For example, by-products of olive processing around 10% of the weight of raw material (leaves and waste) that arrives at the olive processing industries are discarded. Studies into finding applications for the by-products produced from the olive oil industry are of great interest not only from an environmental point of view but also from an economic and the human health perspective, because the production of functional foods elaborated with natural extracts from *Olea europaea* can be an excellent strategy in the food industry [16].

In the processing of virgin olive oil, the malaxation step and crushing of the olive are the main steps to obtain the paste for separation of the oil. Subsequently, the separation of the oil phase is produced through centrifugation or pressure. The three-phase centrifugation system is the main extraction method used in Mediterranean countries [17]. With this method, three phases are separated: the pomace (solid), the olive oil and the wastewater. Regarding quantities after the olive oil extraction, an effluent (olive mill wastewater) containing the combination of water (from processing and washing the olives) and the water contained in the olive fruit is produced. Between 10 and 30 million m^3^ of wastewater are produced every year [18].

These three phases are rich sources of a polyphenols with a large spectrum of biological activities. Therefore, valuable compounds could be obtained from those materials for the preparation of functional food ingredients and nutraceuticals. The phenolic content in the olive oil accounts for only 2% while the remaining content (98%) is lost in the pomace and olive mill wastewater because hydrolyzed polyphenols are liberated into the brines [19]. Polyphenols from olives may have significant health benefits such as antiatherogenic, antimicrobial, antitumoral, anti-inflammatory, cardioprotective and cytoprotective properties [20,21]. In addition, phenolic compounds of oil wastewater have been demonstrated to have antimicrobial activity [22]. Therefore, there is a growing interest in using by-products from the olive industry in several applications, such as food supplements, functional foods, nutraceuticals and pharmaceutical products [23,24].

**Figure 1:**
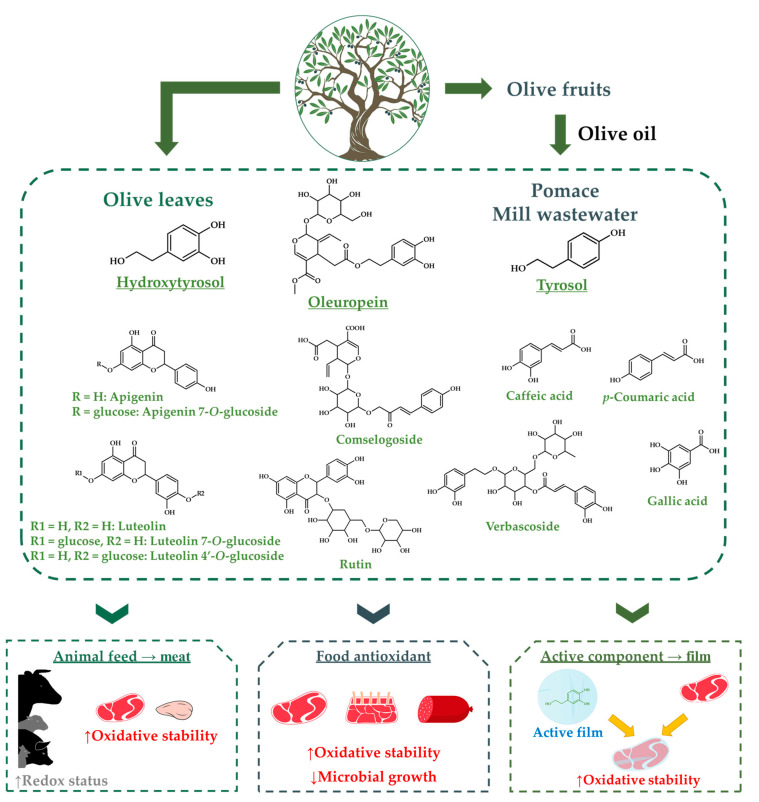
Schematic representation of the strategies to use polyphenol-rich extracts from *Olea europaea* to improve the quality and preservation of meat and meat products.

In addition to these aspects, it is also relevant to consider the concerns among consumers about the presence of synthetic additives widely applied in meat products processing, such as nitrites, sulphites, butylated hydroxytoluene (BHT) or butylated hydroxyanisole (BHA), and their willingness to buy healthier and safer meat alternatives (clean label products) [25,26,27,28,29,30]. Based on this health concern, the reduction and/or replacement of these synthetic preservatives by natural extracts from plants have been receiving great attention from researchers and professionals of the meat industry [26,31,32,33,34,35,36,37,38,39,40]. One of the research fields is to study the different strategies to produce and select natural extracts from olive industry by-products that do not modify sensory parameters and improve the preservation of meat and meat products. Some possibilities exist in the development of functional meat products in order to facilitate the incorporation of bioactive compounds and/or limit those that can produce harmful effects on the consumer health. There are several strategies to develop functional products using food of animal origin, firstly focused on animal production (endogenous enrichment) and, secondly, on technological systems (exogenous enrichment). Endogenous enrichment of animal origin products can be commonly carried out throughout genetic or nutritional modifications in animal feeding. Alternatively, the exogenous enrichment can be carried out with the direct transformation of the raw material or the formulation of processed animal origin products by incorporating potential functional ingredients or by introducing the active component into a film (Figure 1).

This review aims to discuss the phenolic composition, antioxidant potential and antimicrobial activity of phenolic compounds found in *Olea europaea* leaves, olive pomace and wastewater as well as their application in animal feeding for meat production and the use in meat products. In addition, a second objective is to find a variety of studies that feature designed food products of animal origin free of artificial preservatives and using natural extracts obtained from olive oil industry by-products.

## 2. Phenolic Profile of *Olea europaea* Leaves, Olive Pomace and Wastewater

### 2.1. Phenolic Profile of Olive Mill Wastewater

Olive mill wastewater is a malodorous acidic liquid (pH 5–5.5) with a strong smell of olive oil and a colour ranging from violet to dark brown [41]. This olive processing by-product is a source of natural antioxidants (especially polyphenols) and other compounds (organic acids, potassium, protein, sugars, phosphatic salts, and other component in stable emulsion state) that end up in the wastewaters [42,43]. Table 1 shows the methods applied to separate, identify and quantify the phenolic compounds found in olive mill wastewater as well as leaves and pomace.

Phenolic components of olive mill wastewater include oleuropein aglycon derivatives, quercetin, luteolin 7-glucoside, and phenolic alcohols [44]. The phenolic compounds identified in this residue include hydroxytyrosol as the major component (66.5%), together with tyrosol, caffeic acid, *p*-coumaric acid, homovanillic acid, protocatechuic acid, 3,4-dihydroxymandelic acid, vanillic acid, and ferulic acid [45]. In addition, β-hydroxyverbascoside, isoverbascoside and verbascoside have been described in olive mill wastewater [46].

### 2.2. Phenolic Profile of Olea europaea Leaves

Olive leaf is a potential renewable, abundant, and inexpensive source of biophenols [47]. The phenolic profile of olive leaves is affected by several agronomical factors, such as geographical origin, degree of ripeness, leaf age and moisture content; and by technological parameters employed for extraction such as solvent type, preliminary preparations, solvent composition, particle size, extraction temperature, extraction time, pH and pressure [48].

Oleuropein is the major phenolic compound in olive leaves, representing 9% of total leaf weight (dry matter) [49]. In addition, Pereria et al. [50] reported that luteolin 7-*O*-glucoside, apigenin 7-*O*-glucoside, and luteolin 4′-*O*-glucoside are among the main polyphenolics in olive leaves. A similar study indicated the presence of glucoside derivatives of luteolin, hydroxytyrosol, verbascoside and apigenin [51]. Among the phenolic acids found in olive leaves, caffeic, *p*-coumaric, chlorogenic, vanillic and homovanillic acid were also detected [52]. Flavonoids were also detected in olive leaves, such as diosmetin, rutin, quercetin, hesperidin, apigenin 7-*O*-rutinoside, apigenin 7-*O*-glucoside, luteolin 7-*O*-glucoside, luteolin, luteolin 7-*O*-rutinoside and luteolin 4-*O*-glucoside [53].

### 2.3. Phenolic Profile of Olive Pomace

Olive pomace is the major residue of oil processing and a rich source of polyphenols [54]. Among the main polyphenols present in olive pomace are hydroxytyrosol and comselogoside that represent ≈79% and in a lower proportion by tyrosol (3.4 mg/100 g) [55]. However, due to its high phenolic content, olive pomace is also considered phytotoxic [56]. Therefore, the use of environmentally friendly solvents and the development of eco-friendly technologies are mandatory to maximize the extraction of bioactive compounds in olive pomace [57].

Olive pomace contains the majority (98%) of phenolics found in olive fruit [58]. Due to chemical transformations that occur during olive pomace storage, the free forms of tyrosol, oleuropein or hydroxytyrosol can be found in olive pomace together with different analogues [56]. Nunes et al. [54] reported that the major compounds identified in olive pomace are distributed as follows: hydroxytyrosol, comselogoside, tyrosol, oleoside riboside. They are distributed as lignans, phenolic alcohols, secoiridoids and derivatives groups [56,59].

## 3. Antioxidant and Antimicrobial Activity of *Olea europaea* Polyphenols

### 3.1. Antioxidant Activity In Vitro

Characterizing the antioxidant activity in extracts is a challenging task that involves more than one method [61]. This condition is derived from the multiple mechanisms associated with antioxidant effect. In food samples, the main mechanisms that take place to explain the delaying of the progression of oxidative reactions are the hydrogen atom transfer (HAT) and single electron transfer (SET) in free radicals [61,62].

In this sense, several methods have been proposed to characterize the mechanisms involved in the antioxidant activity of natural extracts. In the case of SET assays, some of the most widely applied tests are cupric reducing antioxidant capacity (CUPRAC), ferric reducing ability of plasma (FRAP), 2,2-diphenyl-1-picrylhydrazyl (DPPH), and 2,2′-azino-bis(3-ethylbenzothiazolline-6-sulfonic acid) (ABTS). Particularly for HAT assays, oxygen radical absorbance capacity (ORAC), β-carotene bleach assay, and inhibition of lipoperoxidation can be cited as tests commonly applied [61,62].

The different parts of *Olea europaea* plant contain antioxidant compounds, especially the by-products generated from the processing of its fruits as indicated by screening methods (Table 2). The capacity to scavenge radicals, evaluated by DPPH and ABTS radical assays, have been applied by several researchers to characterize the antioxidant activity of olive processing by-products [15,63,64,65,66,67]. It is also relevant mentioning that the characterization of antioxidant activity of *Olea europaea* extracts by CUPRAC [66] and ORAC [68] assays were also reported in literature. These results support the hypothesis that the antioxidant found in *Olea europaea* plant can scavenge free radicals by different mechanisms (SET and HAT) and delay oxidative reactions.

Another important aspect related to the antioxidant activity of *Olea europaea* extract is the influence of sample preparation (prior to extraction stage) and extracting conditions. For instance, the study performed by Şahin et al. [15] explored the influence of microwave (MW) drying conditions on the antioxidant activity of *Olea europaea* leaves extracts. According to these authors, a significant effect was obtained by using different levels of MW power and solid mass as well as drying time [15]. A related study reported the effect of drying method (freeze vs. hot air vs. drum drying) and conditions (high- vs. low-speed drum drying) on the antioxidant activity of olive oil pomace [65]. Although all drying methods reduced the antioxidant activity in comparison to extract obtained from fresh pomace, the use of drum drying at low speed caused the lowest reduction in antioxidant activity of extract in comparison to other drying methods.

Once the olives are collected and properly prepared for oil extraction, the processing is carried out and generates residues rich in antioxidant compounds. In the case of alpeorujo, a recent study evaluated the influence of steam treatment (80 °C or 120 °C for 60 min or 160 °C for 30 min) and bleaching in the antioxidant capacity of alpeorujo ethanolic extract [68]. According to the authors, the highest antioxidant activity was obtained using steam treatment at 80 °C for 60 min without bleaching the alpeorujo (around 400 μmol TE/g).

In the case of solvent effect on the antioxidant activity of olive oil by-products extract, it was indicated that hydroethanolic [63], hydromethanolic [69] and acidified water (5% HCl) [64] are interesting solvents to obtain extracts with high antioxidant activity from pomace. In the case of peels, acidified water (5% HCl) was the most efficient solvent to extract antioxidant compounds. Particularly for seeds, the use of boiling water, acidified water (1 and 5% HCl) and 50% hydroethanolic solution were more efficient to extract antioxidants than cold water [64].

The proportion of solid and solvent also plays an important role in the recovery of antioxidant from olive oil pomace. This outcome was reported by Goldsmith et al. [66], who indicated that 2 g:100 mL (water) was the optimum proportion to improve the extraction of antioxidants from olive oil pomace. A similar experiment carried out by De Bruno et al. [63] with the same olive oil by-product indicated that using a ratio of 1 g:4 mL for *Carolea* cultivar and 1 g:2 mL for *Ottobratica* cultivar produced extracts with high antioxidant capacity.

Another relevant variable that affects the antioxidant activity of olive oil by-products extracts is extraction time. This variable was studied by Goldsmith et al. [66] in olive oil pomace. According to the authors, increasing extraction time from 45 or 60 to 75 min was associated with a higher antioxidant activity. Accordingly, De Bruno et al. [63] indicated that increasing extraction from 30 or 60 to 120 min improved the antioxidant activity of olive oil pomace extracts. In the case of leaves, a study reported a shorter extraction time (83 s) [15] in comparison to these aforementioned studies using pomace. A study exploring the influence of ultrasound (US) power on the extraction of antioxidants from olive oil pomace indicated that the highest values were obtained by 250 W in comparison to less intense treatments (100 and 150 W) [66].

It is worth mentioning that wastewater also contains compounds with antioxidant activity. According to Akretche et al. [67], this residue is rich in polyphenols (especially hydroxytyrosol and tyrosol) and can scavenge free radicals after a simple stage preparation (centrifugation at 15,000 g for 1 h at 4 °C). Collectively, the antioxidant activity of compounds found in *Olea europaea* has free radical scavenging activity, a capacity to reduce transition metals (CUPRAC assay). Moreover, the extracting conditions (drying of raw material, solvent composition, solid/solvent ratio, extraction time as well as the number of extractions) can influence the extraction of phenolic compounds.

### 3.2. Antioxidant Activity In Vivo in Meat-Producing Animals

The aerobic respiration, oxidative metabolism, and biochemical processes generate low levels of reactive species in aerobic organisms. In normal conditions, reactive species are converted into less reactive molecules by the action of both endogenous and exogenous antioxidants [70,71,72]. In terms of enzymatic antioxidants, three enzymes are involved: superoxide dismutase (SOD), catalase (CAT), and glutathione peroxidase (GPX). Particularly for SOD, this enzyme catalyzes the decomposition of superoxide anion to hydrogen peroxide (H_2_O_2_) and oxygen (O_2_). CAT is an enzyme that forms water and oxygen from H_2_O_2_ as well as reducing lipid hydroperoxides (ROOH) into lipid alcohol (ROH), water and a deprotonated H donor. In the case of GPX, ROOH are reduced to ROH consuming glutathione (GSH) [70].

In the case of non-enzymatic antioxidants, many molecules have been included in this group such as bilirubin, glutathione (GSH), melatonin, metal binding proteins, polyamines, reduced coenzyme Q, thiols, and uric acid (UA) [71,72]. These molecules are directly involved with inactivation of oxidizing compounds as well as intermediate products of oxidative reactions with potential impact on redox balance [72].

Although the endogenous antioxidant defenses are constantly balancing the redox status towards the homeostasis, additional antioxidant protection (by means of exogenous antioxidants) is of great value to assist the natural defense against reactive species, such as polyphenols, vitamin E, and alpha-tocopherol [72,73].

Another relevant approach to characterize the redox status is the evaluation of formation of oxidation products, such as those from lipids (thiobarbituric acid reactive substances—TBARS) and proteins (carbonyl formation) [72]. Collectively, the levels of endogenous and exogenous antioxidants as well as the accumulation of oxidation products provide a comprehensive view of redox status in an aerobic organism [72]. In this line of thought, studies have been carried out on the influence of natural antioxidants from *Olea europaea* parts in the redox status of animals used for meat products (Table 3).

A relevant example of the protective effect of *Olea europaea* parts against oxidative stress is the study carried out by Gerasopoulos et al. [74] with pigs. According to the authors, a 2% addition of either retentate or permeate mill wastewater (obtained using a ceramic microfiltration membrane and a resin column, respectively) improved the total antioxidant capacity (TAC) and also reduced the levels of carbonyl and TBARS in the blood of animals fed with the enriched diet. Similarly, Rey et al. [75] indicated that the blood total antioxidant capacity (TAC) and GSH level increased in pigs fed with oleuropein (one of the phenolic compounds found in the leaves of *Olea europaea*).

In the case of cattle, a study indicated that calves consuming a feed with 15% olive oil pomace had higher levels in terms of antioxidant capacity, uric acid and catalase activity than animals in the control diet. The oxidation markers in the blood of animals fed with the pomace diet were reduced and no effect on GPx was reported by the authors [76]. The in vivo antioxidant effect of *Olea europaea* polyphenols was also studied in goats, such as those reported by Hukerdi et al. [77]. According to the authors, a concentration-dependent effect of olive leaves (7.5 and 15% of feed) in the redox status increased TAC and reduced TBARS level in plasma of Mahabadi goats.

Likewise, some recent studies reported that poultry redox status can be improved by the use of *Olea europaea* polyphenols. For instance, Ahmed et al. [78] indicated that using 50.0, 100.0, or 150 mg oleuropein/kg feed increased the total antioxidant capacity and SOD activity as well as reduced the TBARS levels in the blood of Bandarah chickens. A related study indicated that higher antioxidant activity and reduced levels of carbonyls and TBARS in the plasma of Hubbard chickens were obtained from animals fed with retentate and permeate (2%) of olive mill wastewater than with chicken feed with control diet [79]. Additionally, the authors indicated that the improvement of redox status was observed throughout the supplementation period. Similarly, Saleh et al. [80] reported a significant reduction in TBARS levels in broiler chickens fed with olive cake meal (2 and 4%) for 35 days.

Two recent studies with Japanese quail (*Coturnix coturnix japonica*) fed with olive pulp from oil extraction and oleuropein indicated a significant increase in the antioxidant status. In the case of olive pulp (50 and 100 g/kg), both GSH and GSR levels were improved but no significant effect was reported for UA and TBARS levels in the blood after 6 weeks of supplemented diet [81]. Likewise, the addition of oleuropein (200 mg/kg) was also associated with increased antioxidant capacity and reduced oxidative stress in the liver of Japanese quail [82].

In terms of assessment, most of the studies reported using blood samples. A large body of evidence supports the importance of measuring the molecules from blood samples (such as CAT, GPx, GSH, SOD, and TBARS) due to their correlation with the redox status in tissues [83]. In the case of the present review, the studies performed by Gerasopoulos et al. [74] with pigs, Hukerdi et al. [77] with goats, and Gerasopoulos et al. [79] with chicken indicated a simultaneous increase in antioxidant potential in the plasma and muscle.

Taking into account the antioxidant defenses against oxidative stress, the use of natural sources in animal feeding is an important strategy to improve the antioxidant status in pigs, cattle, goat, chicken, and quail used for meat production and to strengthen the functional role (in animals) of the *Olea europaea* plant as source of bioactive polyphenols.

### 3.3. Antimicrobial Activity In Vitro

The addition of natural preservatives in meat and meat products is part of the actions carried out to develop foods in accordance with consumers’ trends [84]. Among the several candidates, phenolic compounds stand out as a relevant group due to the diversity of compounds and effectiveness against several spoilage and pathogenic microorganisms [85]. In the case of *Olea europaea* polyphenols, recent studies indicated that the pomace, leaves, and commercial extracts rich in polyphenols have antimicrobial activity against both Gram-positive and Gram-negative bacteria (Table 4). This antimicrobial activity is dependent on the composition of the extract, as indicated by Wahdan and Taha [69], who reported significant differences in the inhibition zone of both *Escherichia coli* and *Staphylococcus aureus* exposed to pomace extracts obtained using acetone or 70% methanol solution. Larger inhibition zones were obtained using the hydromethanolic extract.

In this line of thought, Friedman et al. [86] compared the antimicrobial effect of Hidrox-12 (a freeze-dried extract from olive juice) with hydroxytyrosol and indicated that a greater antimicrobial effect was obtained using the isolated compounds rather than the extract against *Staphylococcus aureus*. Furthermore, the comparison of antimicrobial activity (*Escherichia coli* and *Salmonella enterica*) between hydroxytyrosol and oleuropein was recently explored by Peng et al. [87]. According to the authors, the lower minimum inhibitory concentration (MIC) values were obtained against both bacteria using hydroxytyrosol rather than oleuropein.

In the case of oleuropein, contrasting results have been reported in terms of the effect against Gram-negative and Gram-positive bacteria. For instance, the study carried out by Dominciano et al. [90] evaluated the antimicrobial effect of oleuropein and reported that the inhibition zone obtained against *E. coli* was smaller than that obtained for *S. aureus*. Similarly, Yuan et al. [92] obtained the same result using an oleuropein extract against the same bacteria. Conversely, another experiment carried out by Dominciano et al. [91] indicated no significant difference between inhibition zones among *Listeria monocytogenes*, *S. aureus* and *E. coli*.

Although differences in the antimicrobial activity have been reported, both hydroxytyrosol [86,87,88,89] and oleuropein [90,91,92] play a central role in the antimicrobial activity of *Olea europaea* polyphenols. Particularly for hydroxytyrosol, the antimicrobial effect in different bacteria was tested against the growth of both Gram-negative (*Aeromonas hydrophila*, *E. coli*, *Erwinia carotovora*, *Klebsiella pneumoniae*, *Pseudomonas aeruginosa*, *Salmonella typhimurium*, *Shigella sonnei*, *S. aureus*, and *Yersinia enterocolitica*) and Gram-positive bacteria (*Kocuria rhizophila*, *L. monocytogenes*, and *Pediococcus acidilactici*). The authors indicated that MIC values ≥1000 μg/mL were obtained for the majority of tested microorganisms for both groups. A similar outcome was reported by Techathuvanan et al. [89] using a commercial extract (Hidrox 10, 46% of hydroxytyrosol) against *E. coli* O157:H7, *Salmonella enterica* serovar Enteritidis, *Enterobacter aerogenes*, *Bacillus cereus*, *S. aureus*, and *L. monocytogenes*. The MIC values obtained for the tested microorganisms ranged between 1400 and 5200 mg/L. Moreover, lower MIC values were obtained for Gram-positive (*Bacillus cereus*, *S. aureus*, and *L. monocytogenes*) then for Gram-negative (*E. coli* O157:H7, *S. enterica* serovar Enteritidis, *Enterobacter aerogenes*) bacteria.

The higher effectiveness of *Olea europaea* polyphenols against bacteria has been attributed to multiple mechanisms. The study performed with a polyphenol-rich extract (mainly composed of hydroxytyrosol) from olive oil processing in *B. cereus* indicated that the bacteria exposed to this extract displayed reduced intracellular ATP and bacterial protein content, depolarized cell membrane and also poor capacity to retain intracellular components [93].

Particularly for the effect of *Olea europaea* polyphenols on the intracellular ATP content of bacteria, Amini et al. [94] explored in detail the relation between DHPG, hydroxytyrosol, tyrosol, and oleuropein with ATP synthase (an enzyme directly involved in the generation of energy in the form of ATP) in *E. coli*. The authors observed that after polyphenols bind to the polyphenol binding pocket of ATP synthase, the activity of this enzyme is reduced, which affects microbial metabolism and eventually leads to death. Collectively, the studies carried out to characterize and understand the impact of *Olea europaea* polyphenol-rich extracts and the major compounds (especially hydroxytyrosol and oleuropein) on bacteria strengthened their use as antimicrobial agents with potential application as preservatives in food products.

## 4. Use of Phenolic Compounds Obtained from *Olea europaea* in Meat Production and Products

### 4.1. Animal Feeding and Meat Quality

The consumption of a polyphenol-rich diet by meat-producing animals can influence the oxidative status and stability of fresh meat during storage (Table 5). Regarding the effect on the fresh meat of lambs, Hamdi et al. [95] indicated that on the oxidative status (measured by the lipid oxidation level and the activity of SOD, GPx, and CAT) of the *longissimus lumborum* obtained from animals fed with olive oil cake no significant affect was observed in comparison to animals in the control group.

Conversely, studies carried out during the shelf life of goat meat indicate the protection of *Olea europaea* polyphenols against oxidative reactions. This outcome was obtained by Hukerdi et al. [77] using the leaves of *Olea europaea* in the diet of Mahabadi goats. According to the authors, significantly lower values were obtained from the meat of animals fed the supplemented diet in comparison to goats given the control diet during storage (10 days at 4 °C). Similarly, Cimmino et al. [96] noticed that the olive mill wastewater improved the oxidative stability of the meat obtained from Saanen goats (7 days at 4 °C).

In the same line of thought, a recent study carried out on the shelf life of fresh pig meat obtained from animals fed with olive dried pulp displayed higher oxidative stability than the meat from animals in the control diet group during 8 days at 4 °C [97]. Conversely, two studies carried out by the same research group with New Zealand White rabbits indicated that the leaves of *Olea europaea* did not influence the lipid oxidation or the formation of thiols in fresh rabbit meat of [98,99].

Particularly for chicken meat, two recent studies indicated contrasting results in terms of the oxidative status of fresh meat. On one side, Branciari et al. [100] reported a significant increase in the oxidative status in the *pectoralis major* of chickens fed with olive cake (16.5 g/100 g feeding) in comparison to lower concentrations of this residue in the control diet. Similarly, da Silva et al. [101] observed that feeding chickens olive leaves reduced the peroxide and conjugated dienes levels and did not affect the TBARS and carbonyl levels of fresh *pectoralis major*. On the other side, Papadomichelakis et al. [102] noticed a slightly pro-oxidant effect in the meat obtained from chickens in the olive dried pulp-enriched diet in comparison to animals in the control diet.

This difference may be may be explained by auto-oxidation of polyphenols. It is worth mentioning that the values obtained by these authors (below 0.6 mg MDA/kg [102]) are under the threshold range for sensory perception of oxidation in meat (between 0.6 and 2.0 mg MDA/kg) [104,105,106]. In terms of oxidative stability during storage, a recent study carried out by Roila et al. [103] indicated that lipid and protein oxidation in chicken breast were delayed due to inclusion of olive mill wastewater in the diet of chickens.

It is worth mentioning that the meat obtained from animals fed with *Olea europaea* polyphenols was also used to produce patties [101] and sausages [107]. In the case of patties, the inclusion of olive leaves in the diet of chickens (5 and 10 g/kg feeding) led to an inhibition in the accumulation of lipid oxidation and protein oxidation in raw frozen burger for up to 60 days of storage [101]. Additionally, the patties displayed significantly lower levels of mesophilic aerobic bacteria, psychrophilic aerobic bacteria, *Staphylococcus* spp., and Enterobacteriaceae throughout the storage period. A similar outcome was noted in the lipid oxidation of fresh pork sausages produced from the meat of animals fed with olive pomace (25% feeding) [107].

Additionally, a large body of evidence indicated that the use of *Olea europaea* polyphenols in animal feed does not negatively affect other quality indicators (pH, cooking loss, and shear force, for instance) of meat obtained from lambs [95,108,109,110,111], goats [77,96], rabbits [98,99], pigs [97,112], cattle [113], and chicken [100,101,102], in terms of chemical composition, pH, color, shear force, drip loss, cooking loss, or sensory properties. Collectively, *Olea europaea* polyphenols can improve the redox status of fresh meat during storage. Additionally, a minimal or not meaningful impact in the quality and oxidative status of fresh meat were also reported, which supports the use of the *Olea europaea* polyphenols in the production of meat.

### 4.2. Meat Products Quality and Shelf Life

In order to prevent the progression of oxidative reactions and the microbial degradation, two strategies have been explored by researchers: the use of natural extracts/isolated compounds as a food additive [29,114,115] and as active components in coatings and films [116,117,118]. In this line of thought, recent studies have explored the effect of *Olea europaea* polyphenols on fresh and minced meat as well as on meat products such as patties, frankfurters, deep fried cuts, and dry-fermented sausages (Table 6).

Regarding the *Olea europaea* polyphenols as a food additive, a recent study explored the use of leave extract concentration in the oxidative stability of raw minced meat [119]. According to the authors, the highest concentration was the most efficient to delay lipid oxidation and also inhibit the growth of psychrotrophic, *Escherichia coli* O157:H7 and *Salmonella enterica* ser. Enteritidis during the refrigerated storage. Moreover, the samples prepared with the natural extracts also received higher scores than those prepared with the control formulation. A related experiment carried out by Aouidi et al. [120] indicated that the formation of metmyoglobin and the lipid oxidation products during storage was reduced in comparison to the control formulation (without antioxidants) in raw and cooked minced beef. The sensory attributes (color, odor, texture, juiciness, taste, and overall appearance) of both raw and cooked minced beef were not affected by the addition of the extract.

Similar outcomes were obtained from experiments with patties. For instance, the use of wet cake extract improved the antioxidant status of raw lamb patties and also delayed the lipid and protein oxidation (particularly for carbonyl with 200 and 400 mg GAE/kg meat) in a modified atmosphere (70% O_2_/30% CO_2_) and refrigerated storage [121]. The authors also indicated that sensory attributes were affected, particularly lamb odor and flavor, but not the overall liking.

In another related experiment with raw beef patties, a slight inhibition of lipid oxidation was reported during frozen storage [122]. However, a different effect was observed in an experiment with cooked beef patties [123]. Although a slight increase in the antioxidant capacity of the patties was obtained by the authors, no significant effect was observed in lipid oxidation and redness in samples produced with olive cake powder. Conversely, antimicrobial activity during storage was observed in all patties with added olive cake powder during storage.

The protective and preservative effect of *Olea europaea* polyphenols was also reported on sausages. The experiment carried out by Nieto et al. [124] with three different extracts obtained from olive wastewater or leaves indicated that lipid and protein oxidation were delayed in chicken frankfurters. Moreover, the authors also observed that sensory attributes related to oxidation (rancid odor and flavor) were less intense in samples produced with natural extracts than in control sausages. In a further experiment, the same research group observed that color and flavor of chicken frankfurters were better preserved in relation to samples produced without antioxidants [31]. Additionally, the authors also obtained negative correlations between unsaturated fatty acids with both lipid oxidation and rancid odor. Particularly for dry-fermented sausages, a recent experiment showed that lipid oxidation was reduced in samples produced with 500 ppm of olive leaf extract and a slight antimicrobial activity was observed during the ripening period (60 days at 4 °C) [125].

In the case of coatings and films incorporated with the polyphenols, they are used to wrap the meat product or are dispersed in a coating solution (where the meat products are immersed). A relevant example of the capacity of active films produced with *Olea europaea* polyphenols to improve the preservation of fresh meat was reported by Bermúdez-Oria et al. [126]. The pectin-fish gelatin active films produced with two concentrations (0.1 and 0.5%) of hydroxytyrosol and 3,4-dihydroxyphenylglycol (DHPG) inhibited lipid oxidation during the refrigerated storage of fresh beef. According to the authors, DHPG displayed more intense antioxidant activity than hydroxytyrosol in two concentrations tested. A similar outcome was reported for Moudache et al. [127] using a polyethylene film with olive leaves extract. All tested concentrations reduced the loss of redness and the accumulation of lipid oxidation products during the refrigerated storage of fresh pork meat. In contrast, the use of oleuropein as an active component of a coating of fried mutton ribs did not affect the oxidative stability, microbial growth or the sensory attributes during the refrigerated storage [128].

Collectively, the antioxidant and antimicrobial potentials characterized in the previous discussions of *Olea europaea* polyphenols was observed in the preservation of meat and meat products, which supports the use of these compounds. Additionally, none of the studies indicated pro-oxidants or microbial stimulatory effects in meat and meat products, and no significant effects on total and free fatty acids, cooking loss, pH, or chemical composition were reported for minced meat, sausages and deep fried ribs [31,120,124,125,128]. The use of *Olea europaea* polyphenols as a food additive can protect meat products from oxidative reactions and microbial growth during processing [125] and storage [31,119,120,121,122,123,124]. In the case of active coatings and films, the incorporation of polyphenols into films [126,127] can be seen as a relevant approach to extend the shelf life of fresh meat. Conversely, more studies of coating solutions and different meat products are necessary to clarify the use of this strategy.

## 5. Conclusions

*Olea europaea* tree and olive processing by-products are relevant sources of polyphenols that can improve the preservation of meat and meat products. The protection against oxidative reactions; inhibitory activity on spoilage and pathogenic microorganisms; preservation of sensory properties; and minor influences on other quality-related indicators in the meat of different species, meat products and storage conditions are important outcomes that support their use. Further research can aim to improve the information about the bioaccessibility of *Olea europaea* polyphenols in meat-producing animals to obtain cuts with enhanced antioxidant potential, promote their use as food additives to improve the stability of reformulated and functional meat products with a higher proportion of unsaturated fatty acids, and also explore the incorporation into edible and sustainable films and coatings to improve the shelf life of meat products.

## Figures and Tables

**Table 1:** Main phenolic compounds found in *Olea europaea* leaves, olive pomace and wastewater.

Source	Extraction and Filtration	Chromatographic Conditions	Identification and Quantification	Main Compounds	Ref.
**Wastewater** Spain and Italy (mg/100 mL of wastewater)	12 mL with 15 mL of acid water; Extrelut 20 mL cartridge	LiChrosorb RP18 column; 26 °C; eluent A and B: water and acetonitrile; flow rate: 1 mL/min	DAD ^1^ and monitoring at 240, 254, 280, 330, and 350 nm; Mass analyser with gas temperature: 350 °C; flow rate: 10.0 L/min; nebulizer pressure 30 psi; quadrupole temperature 30 °C; and capillary voltage: 350 V	Hydroxytyrosol (3.6–13.1), tyrosol (2.9–4.1), caffeic acid (0.4), dAcO1ag ^4^ (132.4), luteolin 7-*O*-glucoside (0.2–36.6), cinnamic acid derivate (0.4–11.8), and luteolin (0.5–62.3)	[60]
**Wastewater** (g TYE ^2^/L)	PES ^3^ membrane microfiltration (0.05 μm pore), 250 rpm; acidification, defatted and ethyl acetate extraction	Lichrosphere C18 column; mobile phase: acetonitrile/water acidified with acetic acid; flow rate: 0.8 mL/min; injection volume: 20 μl	Standard compounds and retention time	Hydroxytyrosol (2.1–3.8), tyrosol (0.2–2.5), *p*-coumaric acid (0.5–0.8), gallic acid (0.3–0.6), hydroxytyrosol-4-β-glucoside (0.17–0.23), caffeic acid (0.1), and oleuropein aglycone (0.1)	[45]
**Leaves**	1.25 g with 25 mL of methanol; 0.22 μm pore syringe filters	Zorbax SB C18 column; 37 °C; eluent A and B: acetic acid in 2 mM sodium acetate and acetonitrile; flow rate: 1 mL/min; injection volume: 10 μl	DAD with standard compounds and monitoring at 280, 320, 360, and 520 nm	Oleuropein (40.33%), verbascoside (5.68%), luteolin 7-*O*-glucoside (5.05%), apigenin 7-*O*-glucoside (3.13%), hydroxytyrosol (1.82%), and tyrosol (1.76%)	[59]
**Leaves** (mg/kg)	MAE ^4^: 1 g with 8 mL of 80% hydroethanolic solution, 8 min, 200 W	Zorbax Eclipse XDB C18; 10 °C; eluent A and B: 0.1% formic acid and 0.1% formic acid in acetonitrile–water; flow rate: 0.8 mL/min; injection volume: 50 μl	Mass analyser with standard compounds; flow rate: 11 L/min; 300 °C; nebuliser pressure: 35 psi; and capillary voltage: 4000 V	Oleuropein (17,000–25,000), verbascoside (1000–2000), apigenin-7-glucoside (137–260), and luteolin-7-glucoside (127–191)	[53]
**Leaves** (mg/kg)	5 g with 150 mL of boiling water, 30 min	Spherisorb ODS2; eluent A and B: water/formic acid (19:1) and methanol; flow rate: 0.9 mL/min; injection volume: 20 µl	DAD with standard compounds and monitoring at 280, 320, and 350 nm	Oleuropein (26,471), luteolin 7-*O*-glucoside (4209), apigenin 7-*O*-glucoside (2333), luteolin 4′-*O*-glucoside (1356), verbascoside (966), rutin (496), and caffeic acid (220)	[50]
**Pomace**	1 g with 50% hydroethanolic solution; 0.22 μm pore syringe filters	Zorbax SB C18 column; 20 °C; eluent A and B: 1% acetic acid and methanol; flow rate: 1 mL/min	FLD ^5^ with wavelength excitation at 280 nm and emission at 330 nm; DAD and monitoring at 280, 320 and 335 nm; Mass analyser with capillary voltage: 15 V; 325 °C; ass scan range: *m*/*z* 100 to 1000	Hydroxytyrosol (53.78%), comselogoside (25.36%), tyrosol (3.03%), oleside riboside (1.96%), oleuropein derivate (1.65%), and verbascoside derivate (1.61%)	[55]
**Pomace** (mg/kg)	Static–dynamic method: 80% hydroethanolic solution; 200 °C; flow rate: 1 mL/min	Zorbax Eclipse XDB C 18; 10 °C; eluent A and B: 0.1% formic acid and 0.1% formic acid in acetonitrile–water; flow rate: 0.8 mL/min; injection volume: 50 μL	Mass analyser with standard compounds; flow rate: 11 L/min; 300 °C; nebuliser pressure: 35 psi; and capillary voltage: 4000 V	Hydroxytyrosol (332–1631), oleuropein (10–660), verbascoside (10–20), apigenin (8–22), luteolin 7-glucoside (4–14), luteolin (3–22), and apigenin 7-glucoside (0.5–6.3)	[53]
**Pomace** (mg/L)	Pressing and filtration: celite and 0.2 μm pore syringe filters	Acquity C18 BEH column; 35 °C; eluent A and B: water/formic acid (99.5/0.5) and acetonitrile; flow rate: 0.3–0.4 mL/min; injection volume: 1 μL	DAD with standard compounds and monitoring at 280, 330, 360, and 520 nm Mass analyser with standard	Hydroxytyrosol (371), hydroxytyrosol glucoside 1 (165), tyrosol (148), hydroxytyrosol glucoside 2 (88), caffeic acid (68), and *p*-coumaric acid (18)	[56]

^1^ Diode array detector; ^2^ Tyrosol equivalents, ^3^ Polyethersulfone, ^4^ Microwave-assisted extraction, and ^5^ Fluorescence detector.

**Table 2:** Antioxidant activity in vitro of different parts of *Olea europaea* and olive oil by-products.

Source and Cultivar (Origin of Samples)	Extraction Conditions	Antioxidant Activity of Extract	Ref.
Leaves, Gemlik cultivar (Marmara. Turkey)	MW ^1^ power (300, 400, and 500 W), solid mass (1.5, 2.0, and 2.5), and drying time (4, 5, and 6 min), solvent (50% methanol), solid/solvent ratio (1 g:64 mL), time (83 s), and filtration	DPPH ^2^: 25.216 mg TE ^3^/g; *optimum conditions: 459.257 W with 2.085 g sample for 6 min*	[15]
Alpeorujo (Sevilla, Spain)	Steam treatment (80 °C or 120 °C for 60 min or 160 °C for 30 min), precipitation (70% ethanol), bleaching (acetic acid and sodium chlorite), and freeze-drying	ORAC ^4^: 387 µmol TE/g; *optimum conditions: 80 °C for 60 min without bleaching*	[68]
Pomace, Arbequina cultivar (California, USA)	Drying: freeze-drying, hot-air drying (80 °C for 130 min), and high- and low-speed drum drying (62 and 105 and s/revolution), solid/solvent ratio (1 g:26.6 mL), solvent (methanol), and time (20 h)	DPPH: all drying methods reduced antioxidant activity; low-speed drum drying was the most efficient to preserve antioxidant activity	[65]
Pomace, *Carolea* and *Ottobratica* cultivar (Florence, Italy)	Defatting (n-hexane), solvent (80 and 100% ethanol), solid/solvent ratio (1 g:2–5 mL), and time (30, 60, and 120 min)	DPPH: >80% inhibition, *optimum conditions: 30 min, 80% ethanol*, and *1 g:4 mL* (*Carolea* cultivar); 55–70% inhibition, *optimum conditions: 120 min, 80% ethanol*, and *1 g:2 mL* (*Ottobratica* cultivar) ABTS ^5^: 20–30 (*Carolea* cultivar) µmol TE/g, *optimum conditions: 120 min, 80% ethanol*, and *1 g:4 mL;* 30–50 (*Ottobratica* cultivar) µmol TE/g, *optimum conditions: 120 min, 80% ethanol*, and *1 g:2 mL*	[63]
Pomace, peels, and seeds (Diyala, Iraq)	Solvent (cold and hot water, 1 and 5% HCl, and 50% hydroethanolic solution), time (2 days), temperature (RT ^6^), and centrifugation (3000 rpm)	DPPH: IC_50_^7^ 72.6 (pomace, 5% HCl), 71.3 (peels, 5% HCl), and 68.-79.3 (seeds; all solvents except cold water) µg/mL	[64]
Pomace (Sharkia, Egypt)	Drying (50 °C), defatting (n-hexane), solvent (acetone and 70% methanol), solid/solvent ratio (1:10), time (48 h), centrifugation (2000 rpm for 15 min), and freeze-drying	DPPH: 83% inhibition (methanolic extract)	[69]
Pomace, Manzanilla cultivar (Hunter Valley, Australia)	Defatting (hexane), US ^8^ power (100, 150, and 250 W), time (45, 60, and 75 min), solid/solvent ratio (1, 2, and 3 g:100 mL)	CUPRAC ^9^: 73.5 mg TE/g; DPPH: 31.2 mg TE/g; *optimum conditions: 250 W*, *75 min*, and *2 g:100 mL* for both antioxidant assays	[66]
Wastewater (Tizi Ouzou, Algeria)	Centrifugation (15000 g for 1 h at 4 °C)	DPPH: increasing antioxidant activity in the range 0.2–2 g/L (>60–80%)	[67]

^1^ MW: microwave; ^2^ DPPH: 2,2-diphenyl-1-picrylhydrazyl; ^3^ TE: trolox equivalent; ^4^ ORAC: Oxygen radical capacity; ^5^ ABTS: 2,2′-Azino-bis(3-ethylbenzothiazoline-6-sulfonic acid); ^6^ RT: room temperature; ^7^ IC_50_: concentration required to clear 50% DPPH free radicals; ^8^ US: ultrasound; and ^9^ CUPRAC: cupric reducing antioxidant capacity.

**Table 3:** Antioxidant activity in vivo of meat-producing animals fed with *Olea europaea* polyphenols.

Animal	Source	Supplementation Proportion and Time	Antioxidant Effect	Ref.
Landrace × Large White Duroc Pietrain pigs	Mill wastewater retentate and permeate	2% of feeding; 50 days	Increased TAC ^1^; reduced carbonyl and MDA ^2^ levels (plasma); increased GSH ^3^, CAT ^4^, and TAC (muscles and other tissues); reduced carbonyl and MDA levels after 15 and 30 days (muscles and other tissues)	[74]
Large White × Landrace pigs	Oleuropein	96 mg/kg feeding; 35 days	Increased TAC and GSH; no effect in MDA levels (plasma)	[75]
Brown Swiss × Baladi calves	Pomace	15% of feeding; 2 months	Increased TAC and catalase activity; reduced MDA levels; no effect in GPx ^5^ (plasma); increased UA ^6^ (liver)	[76]
Mahabadi goat	Leaves	7.5 and 15% of feeding; 84 days	Increased TAC (plasma and muscle); increased GPx (muscle); reduced MDA (plasma)	[77]
Bandarah chickens	Leaves extract	50.0, 100.0, and 150 mg oleuropein/kg feeding; 24 weeks	Increased TAC and SOD ^7^ activity; reduced MDA levels (plasma)	[78]
Hubbard broiler chickens	Mill wastewater retentate and permeate	2% of feeding; 17, 27, and 37 days	Increased TAC; reduced carbonyl and MDA levels (plasma)	[79]
Broiler chickens	Cake meal	2 and 4% of feeding; 35 days	No effect in UA level; slight reduction in MDA levels (plasma)	[80]
Japanese quail	Pulp	50 and 100 g/kg feeding; 6 weeks	Increased GSH and GSR ^8^; no effect in UA on MDA level (plasma)	[81]
Japanese quail	Oleuropein	200 mg/kg feeding; 2 weeks	Increased total antioxidant status; reduced total oxidative stress (liver)	[82]

^1^ TAC: total antioxidant capacity; ^2^ MDA: malondialdehyde; ^3^ GSH: glutathione; ^4^ CAT: catalase; ^5^ GPx: glutathione peroxidase; ^6^ UA: uric acid; ^7^ SOD: superoxide dismutase; and ^8^ GSR: glutathione reductase.

**Table 4:** Antimicrobial activity in vitro of polyphenol-rich extracts and isolated compounds from *Olea europaea*.

Source	Tested Microorganisms	Antimicrobial Effect	Ref.
Pomace extract (acetonic and hydromethanolic extracts)	*Escherichia coli*, and *Staphylococcus aureus*	Inhibition zone: 10 mm for acetone extract for *E. coli* and *S. aureus*; 14 and 15 mm for hydromethanolic extract for *E. coli* and *S. aureus*	[69]
Hydroxytyrosol (commercial isolate) and Hidrox-12 (commercial extract)	*S. aureus* (ATCC6538; non-MRSA strain)	Dose-dependent effect for both commercial products	[86]
Hydroxytyrosol and oleuropein (fruit or leaves extract)	*E. coli* (EHEC) (ATCC 700927) and *Salmonella enterica* serovar Typhimurium (ATCC 19585)	MIC ^1^ hydroxytyrosol: 0.75 and 0.5 g/L for *E. coli* and *S. enterica*, respectively; MIC oleuropein: 17 and 8 g/L for *E. coli* and *S. enterica*, respectively	[87]
Hydroxytyrosol (commercial isolate)	*Aeromonas hydrophila* CECT 389, *E. coli* CECT 4972, *Erwinia carotovora* CECT 225, *Klebsiella pneumoniae* CECT 143, *Kocuria rhizophila* CECT 4070, *Listeria monocytogenes* CECT 940, *Pediococcus acidilactici* CECT 98, *Pseudomonas aeruginosa* CECT 110, *S. typhimurium* NCTC 1201, *Shigella sonnei* CECT 457, *S. aureus* CECT 794, and *Yersinia enterocolitica* CECT 4315.	MIC: >1000 μg/mL for *P. aeruginosa, Y. enterocolitica*, and *S. typhimurium*, *A. hydrophila*, and *L. monocytogenes*; 1000 μg/mL for *E. carotovora*, *K. pneumoniae*, *S. sonnei*, *P. acidilactici*, and *K. rhizophila*; 400 μg/mL for *E. coli* and *S. aureus*	[88]
Hidrox 10X (commercial extract)	*E. coli* O157:H7 ATCC BAA-1882, *S. enterica* serovar Enteritidis ATCC BAA-1045, *Enterobacter aerogenes* ATCC 13048, *Bacillus cereus* F4433/73, *S. aureus* ATCC 25923, and *L. monocytogenes* ATCC 19111	MIC: 1400–2200 mg/L for *B. cereus*, 2000–2500 mg/L for *S. aureus*, 2200–2600 mg/L for *L. monocytogenes*, 2800–3600 mg/L for *S. enterica*, 3600–4200 mg/L for *E. coli*, and 4800–5200 mg/L for *E. aerogenes*	[89]
Oleuropein (commercial isolate)	*E. coli* (ATCC 25922) and *S. aureus* (ATCC 25923); count 10^8^ Concentration: 4.0 mg/mL	Inhibition zone: 7.3 mm for *E. coli* and 10.0 mm for *S. aureus*	[90]
Oleuropein (commercial isolate)	*L. monocytogenes* (ATCC 7644), *S. aureus* (ATCC 25923) and *E. coli* (ATCC 25922)	MIC: 0.200 mg/mL for all microorganisms	[91]
Oleuropein (commercial extract)	*E. coli* (CMCC 44102) and *S. aureus* (CMCC 26003)	Inhibition zone: 10.2–17.9 mm against *E. coli* and 10.5–24.8 mm against *S. aureus*	[92]

^1^ MIC: minimum inhibitory concentration.

**Table 5:** Antioxidant of *Olea europaea* polyphenols as feed supplements in fresh meat.

Animal (Muscle)	Source	Animal Treatments	Point(s) of Assay	Effect on Meat Quality	Ref.
Barbarine lambs (*longissimus lumborum*)	Cake	280 g/day; feed until achieve 33 kg live weight	In fresh meat	No effect on lipid oxidation, CAT ^1^, GSH ^2^, and SOD ^3^	[95]
Mahabadi goats (*longissimus lumborum*)	Leaves	7.5 and 15% feeding; 84 days of feeding	During 10 days at 4 °C	Slowed the increase of MDA ^4^ levels up to 10 days	[77]
Saanen goat (*longissimus thoracis et lumborum*)	Mill wastewater	3.2 mg/day; 78 days	During 7 days at 4 °C	Reduced lipid oxidation during storage	[96]
New Zealand White rabbits (*longissimus thoracis et lumborum*)	Leaves	10% feeding; 35 days	In fresh meat	No effect on lipid oxidation	[98]
New Zealand White rabbits (*longissimus dorsi*)	Leaves	10% feeding; 35 days	In fresh meat	No effect on lipid oxidation and thiol; slight increase in carbonyl content	[99]
PIC Landrace × PIC Large white pigs (*longissimus thoracis*)	Dried pulp	50 g/kg feeding; 30 days	During 8 days at 4 °C	Significant antioxidant effect in the first day of storage	[97]
Ross 308 chickens (*pectoralis major*)	Cake	8.25 and 16.5 g/100 g feeding; 20 days	In fresh meat	Increased the antioxidant capacity and reduced lipid oxidation using 16.5 g/100 g	[100]
Cobb chickens (*pectoralis major*)	Leaves	5 and 10 g/kg feeding; 41 days	In fresh meat	Reduced primary lipid oxidation products	[101]
Cobb 500 chickens (*pectoralis major*)	Dried pulp	25 + 50, 50, and 50 + 80 g/kg feeding in grower and finisher diets; 31 days	In fresh meat	Slight prooxidant effect	[102]
Ross 308 (*pectoralis major*)	Mill wastewater	4.8 and 9.9%; 20 days	During 7 days at 4 °C	Slowed both lipid and protein oxidation	[103]

^1^ CAT: catalase; ^2^ GSH: glutathione; ^3^ SOD: superoxide dismutase; and ^4^ MDA: malondialdehyde.

**Table 6:** Antioxidant and antimicrobial activity of polyphenol-rich extracts from *Olea europaea* and isolated compounds in meat and meat products.

Meat or Meat Product	Source/Isolated Compound and Treatments	Point(s) of Assay	Effect in Quality and Shelf Life	Ref.
Raw minced beef	Leaves; 1 and 5% (*v*/*w*)	Stored for 7 days at 7 °C	5% extract prevented psychrotrophic growth and slight inhibition of *Escherichia coli* O157:H7 and *Salmonella enterica* ser. Enteritidis; slowed lipid oxidation; better scores in sensory analysis	[119]
Raw and cooked minced beef	Leaves; 100 and 150 μg phenols/g meat	Stored for 12 days at 4 °C	Reduced the formation of metmyoglobin and lipid oxidation; no effect on sensory attributes	[120]
Raw lamb patties	Wet cake; 100, 200, and 400 mg GAE/kg meat (modified atmosphere)	Stored for 9 days at 4 °C	Inhibited lipid and protein oxidation (carbonyl; no effect for thiol); reduced lamb odor, fish odor, lamb flavor, fish flavor; increased odd odor and flavor	[121]
Raw beef patties	Oleuropein; 0.25, 0.5, and 0.75%	Stored for 6 months at −12 °C	Slight inhibition in lipid oxidation	[122]
Cooked beef patties	Cake; 2, 4 or 6% (*w*/*w*)	Stored for 7 days at 4 °C	Slight increase in antioxidant activity of patties; no effect on lipid oxidation and redness; inhibited microbial growth; better preservation of sensory properties during storage	[123]
Chicken frankfurters	Wastewater or leaves; 50 ppm	Stored for 21 days at 4 °C	Reduced lipid and protein oxidation, rancid odor and flavor	[124]
Chicken frankfurters	Wastewater; 50 ppm	Stored for 21 days at 4 °C	Better preservation of sausage flavor and color; negative correlations between fatty acids and oxidation markers	[31]
Dry-fermented sausage	Leaves; 125, 250 and 500 ppm	Ripening: 60 days at 4 °C	500 ppm reduced lipid oxidation; no effect on color; slight antimicrobial activity	[125]
Fresh beef	Hydroxytyrosol and DHPG ^1^; 0.1 and 0.5% (*w*/*w*) in pectin-fish gelatin film	Stored for 6 days at 4 °C	Reduced the progression of lipid oxidation products, DHPG was more efficient than hydroxytyrosol	[126]
Fresh pork meat	Leaves; 2, 5, 10, and 15% in polyethylene film	Stored for 16 days at 4 °C	Reduced lipid oxidation and the loss of redness	[127]
Fried mutton ribs	Oleuropein; 0.3, 0.6, and 0.9% (*w*/*v*) in coating solution with glycerite	Stored for 21 days at 4 °C	No significant effect on MDA level, microbial growth, and sensory properties	[128]

^1^ DHPG: 3,4-dihydroxyphenylglycol.

## References

[1] Ray N.B., Lam N.T., Luc R., Bonvino N.P., Karagiannis T.C. (2015). Cellular and molecular effects of bioactive phenolic compounds in olives and olive oil. Olive and Olive Oil Bioactive Constituents.

[2] International Olive Oil Council Economic Affairs & Promotion Unit—International Olive Council. https://www.internationaloliveoil.org/what-we-do/economic-affairs-promotion-unit/#figures.

[3] Zamuz S., Purrinos L., Tomasevic I., Dominguez R., Brnčić M., Barba F.J., Lorenzo J.M. (2020). Consumer acceptance and quality parameters of the commercial olive oils manufactured with cultivars grown in Galicia (NW Spain). Foods.

[4] López-López A., Sánchez-Gómez A.H., Montaño A., Cortés-Delgado A., Garrido-Fernández A. (2019). Sensory characterisation of black ripe table olives from Spanish Manzanilla and Hojiblanca cultivars. Food Res. Int..

[5] Guo Z., Jia X., Zheng Z., Lu X., Zheng Y., Zheng B., Xiao J. (2018). Chemical composition and nutritional function of olive (*Olea europaea* L.): A review. Phytochem. Rev..

[6] Carlos S., De La Fuente-Arrillaga C., Bes-Rastrollo M., Razquin C., Rico-Campà A., Martínez-González M.A., Ruiz-Canela M. (2018). Mediterranean diet and health outcomes in the SUN cohort. Nutrients.

[7] Gavahian M., Mousavi Khaneghah A., Lorenzo J.M., Munekata P.E.S., Garcia-Mantrana I., Collado M.C., Meléndez-Martínez A.J., Barba F.J. (2019). Health benefits of olive oil and its components: Impacts on gut microbiota antioxidant activities, and prevention of noncommunicable diseases. Trends Food Sci. Technol..

[8] (1991). European Commission Commission Regulation (EEC) No 2568/91 of 11 July 1991 on the characteristics of olive oil and olive-residue oil and on the relevant methods of analysis. Off. J. Eur. Communities.

[9] United States Department of Agriculture Olive Oil and olive-Pomace Oil Grades and Standards—Agricultural Marketing Service. https://www.ams.usda.gov/grades-standards/olive-oil-and-olive-pomace-oil-grades-and-standards.

[10] International Olive Council (2019). Trade Standard Applying to Olive Oils and Olive Pomace Oils.

[11] European Comission (2020). The EU Food Fraud Network and the Administrative Assistance and Cooperation System Health and Food Safety.

[12] Bajoub A., Bendini A., Fernández-Gutiérrez A., Carrasco-Pancorbo A. (2018). Olive oil authentication: A comparative analysis of regulatory frameworks with especial emphasis on quality and authenticity indices, and recent analytical techniques developed for their assessment. A review. Crit. Rev. Food Sci. Nutr..

[13] Gómez-Caravaca A.M., Maggio R.M., Cerretani L. (2016). Chemometric applications to assess quality and critical parameters of virgin and extra-virgin olive oil. A review. Anal. Chim. Acta.

[14] Alirezalu K., Pateiro M., Yaghoubi M., Alirezalu A., Peighambardoust S.H., Lorenzo J.M. (2020). Phytochemical constituents, advanced extraction technologies and techno-functional properties of selected Mediterranean plants for use in meat products. A comprehensive review. Trends Food Sci. Technol..

[15] Şahin S., Elhussein E., Bilgin M., Lorenzo J.M., Barba F.J., Roohinejad S. (2018). Effect of drying method on oleuropein, total phenolic content, flavonoid content, and antioxidant activity of olive (*Olea europaea*) leaf. J. Food Process. Preserv..

[16] Sahin S., Samli R., Birteks Z Tan A.S., Barba F.J., Chemat F., Cravotto G., Lorenzo J.M. (2017). Solvent-free microwave-assisted extraction of polyphenols from olive tree leaves: Antioxidant and antimicrobial properties. Molecules.

[17] Souilem S., El-Abbassi A., Kiai H., Hafidi A., Sayadi S., Galanakis C.M. (2017). Olive oil production sector: Environmental effects and sustainability challenges. Olive Mill Waste: Recent Advances for Sustainable Management.

[18] Doula M.K., Moreno-Ortego J.L., Tinivella F., Inglezakis V.J., Sarris A., Komnitsas K. (2017). Olive mill waste: Recent advances for the sustainable development of olive oil industry. Olive Mill Waste: Recent Advances for Sustainable Management.

[19] Rodis P.S., Karathanos V.T., Mantzavinou A. (2002). Partitioning of olive oil antioxidants between oil and water phases. J. Agric. Food Chem..

[20] Zbakh H., El Abbassi A. (2012). Potential use of olive mill wastewater in the preparation of functional beverages: A review. J. Funct. Foods.

[21] Cicerale S., Conlan X.A., Sinclair A.J., Keast R.S.J. (2009). Chemistry and health of olive oil phenolics. Crit. Rev. Food Sci. Nutr..

[22] Obied H.K., Bedgood D.R., Prenzler P.D., Robards K. (2007). Bioscreening of Australian olive mill waste extracts: Biophenol content, antioxidant, antimicrobial and molluscicidal activities. Food Chem. Toxicol..

[23] El S.N., Karakaya S. (2009). Olive tree (Olea europaea) leaves: Potential beneficial effects on human health. Nutr. Rev..

[24] Granato D., Barba F.J., Bursać Kovačević D., Lorenzo J.M., Cruz A.G., Putnik P. (2020). Functional foods: Product development, technological trends, efficacy testing, and safety. Annu. Rev. Food Sci. Technol..

[25] Fernandes R.P.P., Trindade M.A., Lorenzo J.M., de Melo M.P. (2018). Assessment of the stability of sheep sausages with the addition of different concentrations of Origanum vulgare extract during storage. Meat Sci..

[26] Fernandes R.P.P., Trindade M.A., Tonin F.G., Lima C.G., Pugine S.M.P., Munekata P.E.S., Lorenzo J.M., de Melo M.P. (2016). Evaluation of antioxidant capacity of 13 plant extracts by three different methods: Cluster analyses applied for selection of the natural extracts with higher antioxidant capacity to replace synthetic antioxidant in lamb burgers. J. Food Sci. Technol..

[27] Fernandes R.P.P., Trindade M.A., Tonin F.G., Pugine S.M.P., Lima C.G., Lorenzo J.M., de Melo M.P. (2017). Evaluation of oxidative stability of lamb burger with *Origanum vulgare* extract. Food Chem..

[28] Lorenzo J.M., González-Rodríguez R.M., Sánchez M., Amado I.R., Franco D. (2013). Effects of natural (grape seed and chestnut extract) and synthetic antioxidants (buthylatedhydroxytoluene, BHT) on the physical, chemical, microbiological and sensory characteristics of dry cured sausage “chorizo”. Food Res. Int..

[29] Nikmaram N., Budaraju S., Barba F.J., Lorenzo J.M., Cox R.B., Mallikarjunan K., Roohinejad S. (2018). Application of plant extracts to improve the shelf-life, nutritional and health-related properties of ready-to-eat meat products. Meat Sci..

[30] Fernandes R.P.P., Trindade M.A., Lorenzo J.M., Munekata P.E.S., de Melo M.P. (2016). Effects of oregano extract on oxidative, microbiological and sensory stability of sheep burgers packed in modified atmosphere. Food Control.

[31] Nieto G., Martínez L., Castillo J., Ros G. (2017). Effect of hydroxytyrosol, walnut and olive oil on nutritional profile of low-fat chicken frankfurters. Eur. J. Lipid Sci. Technol..

[32] Estévez M., Lorenzo J.M. (2019). Impact of antioxidants on oxidized proteins and lipids in processed meat. Encycl. Food Chem..

[33] Martínez L., Castillo J., Ros G., Nieto G. (2019). Antioxidant and antimicrobial activity of rosemary, pomegranate and olive extracts in fish patties. Antioxidants.

[34] Martínez L., Ros G., Nieto G. (2020). Effect of natural extracts obtained from food industry by-products on nutritional quality and shelf life of chicken nuggets enriched with organic Zn and Se provided in broiler diet. Poult. Sci..

[35] Munekata P.E.S., Domínguez R., Campagnol P.C.B.P.C.B., Franco D., Trindade M.A.M.A., Lorenzo J.M.J.M. (2017). Effect of natural antioxidants on physicochemical properties and lipid stability of pork liver pâté manufactured with healthy oils during refrigerated storage. J. Food Sci. Technol..

[36] Pateiro M., Lorenzo J., Vázquez J., Franco D. (2015). Oxidation Stability of Pig Liver Pâté with Increasing Levels of Natural Antioxidants (Grape and Tea). Antioxidants.

[37] Pateiro M., Vargas F.C., Chincha A.A.I.A., Sant’Ana A.S., Strozzi I., Rocchetti G., Barba F.J., Domínguez R., Lucini L., do Amaral Sobral P.J. (2018). Guarana seed extracts as a useful strategy to extend the shelf life of pork patties: UHPLC-ESI/QTOF phenolic profile and impact on microbial inactivation, lipid and protein oxidation and antioxidant capacity. Food Res. Int..

[38] Lorenzo J.M., Munekata P.E.S., Sant’Ana A.S., Carvalho R.B., Barba F.J., Toldrá F., Mora L., Trindade M.A. (2018). Main characteristics of peanut skin and its role for the preservation of meat products. Trends Food Sci. Technol..

[39] de Carvalho F.A.L., Munekata P.E.S., Lopes de Oliveira A., Pateiro M., Domínguez R., Trindade M.A., Lorenzo J.M. (2020). Turmeric (*Curcuma longa* L.) extract on oxidative stability, physicochemical and sensory properties of fresh lamb sausage with fat replacement by tiger nut (*Cyperus esculentus* L.) oil. Food Res. Int..

[40] Pateiro M., Lorenzo J.M.M., Amado I.R.R., Franco D. (2014). Effect of addition of green tea, chestnut and grape extract on the shelf-life of pig liver pâté. Food Chem..

[41] Pierantozzi P., Zampini C., Torres M., Isla M.I., Verdenelli R.A., Meriles J.M., Maestri D. (2012). Physico-chemical and toxicological assessment of liquid wastes from olive processing-related industries. J. Sci. Food Agric..

[42] Saviozzi A., Riffaldi R., Levi-Minzi R., Scagnozzi A., Vanni G. (1993). Decomposition of vegetation-water sludge in soil. Bioresour. Technol..

[43] Paredes C., Cegarra J., Roig A., Sánchez-Monedero M.A., Bernal M.P. (1999). Characterization of olive mill wastewater (alpechin) and its sludge for agricultural purposes. Bioresour. Technol..

[44] Visioli F., Romani A., Mulinacci N., Zarini S., Conte D., Vincieri F.F., Galli C. (1999). Antioxidant and other biological activities of olive mill waste waters. J. Agric. Food Chem..

[45] El-Abbassi A., Kiai H., Hafidi A. (2012). Phenolic profile and antioxidant activities of olive mill wastewater. Food Chem..

[46] Cardinali A., Pati S., Minervini F., D’Antuono I., Linsalata V., Lattanzio V. (2012). Verbascoside, isoverbascoside, and their derivatives recovered from olive mill wastewater as possible food antioxidants. J. Agric. Food Chem..

[47] Özcan M.M., Matthäus B. (2017). A review: Benefit and bioactive properties of olive (*Olea europaea* L.) leaves. Eur. Food Res. Technol..

[48] Şahin S., Bilgin M. (2018). Olive tree (*Olea europaea* L.) leaf as a waste by-product of table olive and olive oil industry: A review. J. Sci. Food Agric..

[49] Kiritsakis K., Kontominas M.G., Kontogiorgis C., Hadjipavlou-Litina D., Moustakas A., Kiritsakis A. (2010). Composition and antioxidant activity of olive leaf extracts from Greek olive cultivars. JAOCS, J. Am. Oil Chem. Soc..

[50] Pereira A.P., Ferreira I.C.F.R., Marcelino F., Valentão P., Andrade P.B., Seabra R., Estevinho L., Bento A., Pereira J.A. (2007). Phenolic compounds and antimicrobial activity of olive (*Olea europaea* L. Cv. Cobrançosa) leaves. Molecules.

[51] Sabry O.M.M. (2014). Beneficial health effects of olive leaves extracts. J. Nat. Sci. Res..

[52] Ryan D., Antolovich M., Herlt T., Prenzler P.D., Lavee S., Robards K. (2002). Identification of phenolic compounds in tissues of the novel olive cultivar Hardy’s Mammoth. J. Agric. Food Chem..

[53] Luján R.J., Capote F.P., de Castro M.D.L. (2009). Temporal metabolomic analysis of *o* -glucoside phenolic compounds and their aglycone forms in olive tree and derived materials. Phytochem. Anal..

[54] Nunes M.A., Pimentel F.B., Costa A.S.G., Alves R.C., Oliveira M.B.P.P. (2016). Olive by-products for functional and food applications: Challenging opportunities to face environmental constraints. Innov. Food Sci. Emerg. Technol..

[55] Nunes M.A., Costa A.S.G., Bessada S., Santos J., Puga H., Alves R.C., Freitas V., Oliveira M.B.P.P. (2018). Olive pomace as a valuable source of bioactive compounds: A study regarding its lipid- and water-soluble components. Sci. Total Environ..

[56] Malapert A., Reboul E., Loonis M., Dangles O., Tomao V. (2018). Direct and Rapid Profiling of Biophenols in Olive Pomace by UHPLC-DAD-MS. Food Anal. Methods.

[57] Galanakis C.M. (2012). Recovery of high added-value components from food wastes: Conventional, emerging technologies and commercialized applications. Trends Food Sci. Technol..

[58] Chanioti S., Tzia C. (2017). Optimization of ultrasound-assisted extraction of oil from olive pomace using response surface technology: Oil recovery, unsaponifiable matter, total phenol content and antioxidant activity. LWT - Food Sci. Technol..

[59] Hayes J.E., Allen P., Brunton N., O’Grady M.N., Kerry J.P. (2011). Phenolic composition and in vitro antioxidant capacity of four commercial phytochemical products: Olive leaf extract (*Olea europaea* L.), lutein, sesamol and ellagic acid. Food Chem..

[60] Mulinacci N., Romani A., Galardi C., Pinelli P., Giaccherini C., Vincieri F.F. (2001). Polyphenolic content in olive oil waste waters and related olive samples. J. Agric. Food Chem..

[61] Granato D., Shahidi F., Wrolstad R., Kilmartin P., Melton L.D., Hidalgo F.J., Miyashita K., van Camp J., Alasalvar C., Ismail A.B. (2018). Antioxidant activity, total phenolics and flavonoids contents: Should we ban in vitro screening methods?. Food Chem..

[62] Karadag A., Ozcelik B., Saner S. (2009). Review of methods to determine antioxidant capacities. Food Anal. Methods.

[63] De Bruno A., Romeo R., Fedele F.L., Sicari A., Piscopo A., Poiana M. (2018). Antioxidant activity shown by olive pomace extracts. J. Environ. Sci. Heal. -Part B Pestic. Food Contam. Agric. Wastes.

[64] Rathi M.H., Turki R.H. (2018). Total phenolic contents and antioxidant activities of olive (*Olea europaea* L.) pomace and their ingredients. J. Al-Nahrain Univ. Sci..

[65] Sinrod A.J.G., Avena-Bustillos R.J., Olson D.A., Crawford L.M., Wang S.C., McHugh T.H. (2019). Phenolics and antioxidant capacity of pitted olive pomace affected by three drying technologies. J. Food Sci..

[66] Goldsmith C.D., Vuong Q. V., Stathopoulos C.E., Roach P.D., Scarlett C.J. (2018). Ultrasound increases the aqueous extraction of phenolic compounds with high antioxidant activity from olive pomace. LWT - Food Sci. Technol..

[67] Akretche H., Pierre G., Moussaoui R., Michaud P., Delattre C. (2019). Valorization of olive mill wastewater for the development of biobased polymer films with antioxidant properties using eco-friendly processes. Green Chem..

[68] Bermúdez-Oria A., Rodríguez-Gutiérrez G., Alaiz M., Vioque J., Girón-Calle J., Fernández-Bolaños J. (2019). Polyphenols associated to pectic polysaccharides account for most of the antiproliferative and antioxidant activities in olive extracts. J. Funct. Foods.

[69] Wahdan K., Taha T. (2018). In vitro antioxidant, antimicrobial and anticancer activities of olive (*Olea europaea* L.) pomace. J. Agric. Chem. Biotechnol..

[70] Matés J.M., Pérez-Gómez C., De Castro I.N. (1999). Antioxidant enzymes and human diseases. Clin. Biochem..

[71] Mirończuk-Chodakowska I., Witkowska A.M., Zujko M.E. (2018). Endogenous non-enzymatic antioxidants in the human body. Adv. Med. Sci..

[72] Pisoschi A.M., Pop A. (2015). The role of antioxidants in the chemistry of oxidative stress: A review. Eur. J. Med. Chem..

[73] Guerra-Araiza C., Álvarez-Mejía A.L., Sánchez-Torres S., Farfan-García E., Mondragón-Lozano R., Pinto-Almazán R., Salgado-Ceballos H. (2013). Effect of natural exogenous antioxidants on aging and on neurodegenerative diseases. Free Radic. Res..

[74] Gerasopoulos K., Stagos D., Petrotos K., Kokkas S., Kantas D., Goulas P., Kouretas D. (2015). Feed supplemented with polyphenolic byproduct from olive mill wastewater processing improves the redox status in blood and tissues of piglets. Food Chem. Toxicol..

[75] Rey A.I., De-Cara A., Calvo L., Puig P., Hechavarría T. (2020). Changes in plasma fatty acids, free amino acids, antioxidant defense, and physiological stress by oleuropein supplementation in pigs prior to slaughter. Antioxidants.

[76] Abdalla E.B., El-Masry K.A., Khalil F.A., Teama F.E., Emara S.S. (2015). Alleviation of oxidative stress by using olive pomace in crossbred (Brown Swiss X Baladi) calves under hot environmental conditions. Arab J. Nucl. Sci. Appl..

[77] Hukerdi Y.J., Nasri M.H.F., Rashidi L., Ganjkhanlou M., Emami A. (2019). Effects of dietary olive leaves on performance, carcass traits, meat stability and antioxidant status of fattening Mahabadi male kids. Meat Sci..

[78] Ahmed M.M., El-Saadany A.S., Shreif E.Y., El-Barbary A.M. (2017). Effect of dietary olive leaves extract (Oleuropein) supplementation on productive, physiological and immunological parameters in Bandarah chickens 2 - During production period. Poult. Sci.

[79] Gerasopoulos K., Stagos D., Kokkas S., Petrotos K., Kantas D., Goulas P., Kouretas D. (2015). Feed supplemented with byproducts from olive oil mill wastewater processing increases antioxidant capacity in broiler chickens. Food Chem. Toxicol..

[80] Saleh A.A., Paray B.A., Dawood M.A.O. (2020). Olive cake meal and bacillus licheniformis impacted the growth performance, muscle fatty acid content, and health status of broiler chickens. Animals.

[81] Abd El-Moneim A.E.M.E., Sabic E.M., Abu-Taleb A.M. (2019). Influence of dietary supplementation of irradiated or non-irradiated olive pulp on biochemical profile, antioxidant status and immune response of Japanese quails. Biol. Rhythm Res..

[82] Sarıca S., Aydın H., Ciftci G. (2017). Effects of dietary supplementation of some antioxidants on liver antioxidant status and plasma biochemistry parameters of heat-stressed quail. Turkish J. Agric. - Food Sci. Technol..

[83] Margaritelis N. V., Veskoukis A.S., Paschalis V., Vrabas I.S., Dipla K., Zafeiridis A., Kyparos A., Nikolaidis M.G. (2015). Blood reflects tissue oxidative stress: A systematic review. Biomarkers.

[84] Aziz M., Karboune S. (2018). Natural antimicrobial/antioxidant agents in meat and poultry products as well as fruits and vegetables: A review. Crit. Rev. Food Sci. Nutr..

[85] Efenberger-Szmechtyk M., Nowak A., Czyzowska A. (2020). Plant extracts rich in polyphenols: Antibacterial agents and natural preservatives for meat and meat products. Crit. Rev. Food Sci. Nutr..

[86] Friedman M., Rasooly R., Do P.M., Henika P.R. (2011). The olive compound 4-hydroxytyrosol inactivates *Staphylococcus aureus* bacteria and staphylococcal enterotoxin A (SEA). J. Food Sci..

[87] Peng M., Zhao X., Biswas D. (2017). Polyphenols and tri-terpenoids from *Olea europaea* L. in alleviation of enteric pathogen infections through limiting bacterial virulence and attenuating inflammation. J. Funct. Foods.

[88] Medina-Martínez M.S., Truchado P., Castro-Ibáñez I., Allende A. (2016). Antimicrobial activity of hydroxytyrosol: A current controversy. Biosci. Biotechnol. Biochem..

[89] Techathuvanan C., Reyes F., David J.R.D., Davidson P.M. (2014). Efficacy of commercial natural antimicrobials alone and in combinations against pathogenic and spoilage microorganisms. J. Food Prot..

[90] Dominciano L.C.C., Lee S.H.I., Santello J.M., De Martinis E.C.P., Corassin C.H., Oliveira C.A.F. (2016). Effect of oleuropein and peracetic acid on suspended cells and mono-species biofilms formed by *Staphylococcus aureus* and *Escherichia coli*. Integr. Food Nutr. Metab..

[91] Dominciano L.C.C., Oliveira C.A.F., Lee S.H., Corassin C.H. (2016). Individual and combined antimicrobial activity of oleuropein and chemical sanitizers. J. Food Chem. Nanotechnol..

[92] Yuan J.J., Tu J.L., Qin F.G.F., Xu Y.J., Li B. (2018). Phenolic composition of oleuropein extract after enzymatic process by HPLC-MS and their antioxidant and antibacterial activities. J. Food Biochem..

[93] Fei P., Xu Y., Zhao S., Gong S., Guo L. (2019). Olive oil polyphenol extract inhibits vegetative cells of *Bacillus cereus* isolated from raw milk. J. Dairy Sci..

[94] Amini A., Liu M., Ahmad Z. (2017). Understanding the link between antimicrobial properties of dietary olive phenolics and bacterial ATP synthase. Int. J. Biol. Macromol..

[95] Hamdi H., Majdoub-Mathlouthi L., Durand D., Thomas A., Kraiem K. (2018). Effects of olive-cake supplementation on fatty acid composition, antioxidant status and lipid and meat-colour stability of Barbarine lambs reared on improved rangeland plus concentrates or indoors with oat hay plus concentrates. Anim. Prod. Sci..

[96] Cimmino R., Barone C.M.A., Claps S., Varricchio E., Rufrano D., Caroprese M., Albenzio M., De Palo P., Campanile G., Neglia G. (2018). Effects of dietary supplementation with polyphenols on meat quality in Saanen goat kids. BMC Vet. Res..

[97] Tsala A., Mpekelis V., Karvelis G., Tsikakis P., Goliomytis M., Simitzis P. (2020). Effects of dried olive pulp dietary supplementation on quality characteristics and antioxidant capacity of pig meat. Foods.

[98] Mattioli S., Machado Duarte J.M., Castellini C., D’Amato R., Regni L., Proietti P., Businelli D., Cotozzolo E., Rodrigues M., Dal Bosco A. (2018). Use of olive leaves (whether or not fortified with sodium selenate) in rabbit feeding: Effect on performance, carcass and meat characteristics, and estimated indexes of fatty acid metabolism. Meat Sci..

[99] Mattioli S., Dal Bosco A., Duarte J.M.M., D’Amato R., Castellini C., Beone G.M., Fontanella M.C., Beghelli D., Regni L., Businelli D. (2019). Use of selenium-enriched olive leaves in the feed of growing rabbits: Effect on oxidative status, mineral profile and selenium speciation of *Longissimus dorsi* meat. J. Trace Elem. Med. Biol..

[100] Branciari R., Galarini R., Giusepponi D., Trabalza-Marinucci M., Forte C., Roila R., Miraglia D., Servili M., Acuti G., Valiani A. (2017). Oxidative status and presence of bioactive compounds in meat from chickens fed polyphenols extracted from olive oil industry waste. Sustainability.

[101] da Silva S.L., Marangoni C., Brum D.S., Vendruscolo R.G., Silva M.S., de Moura H.C., Rampelotto C., Wagner R., de Menezes C.R., Barin J.S. (2018). Effect of dietary olive leaves on the lipid and protein oxidation and bacterial safety of chicken hamburgers during frozen storage. Int. Food Res. J..

[102] Papadomichelakis G., Pappas A.C., Tsiplakou E., Symeon G.K., Sotirakoglou K., Mpekelis V., Fegeros K., Zervas G. (2019). Effects of dietary dried olive pulp inclusion on growth performance and meat quality of broiler chickens. Livest. Sci..

[103] Roila R., Valiani A., Miraglia D., Ranucci D., Forte C., Trabalza-Marinucci M., Servili M., Codini M., Branciari R. (2018). Olive mill wastewater phenolic concentrate as natural antioxidant against lipid-protein oxidative deterioration in chicken meat during storage. Ital. J. Food Saf..

[104] Campo M.M., Nute G.R., Hughes S.I., Enser M., Wood J.D., Richardson R.I. (2006). Flavour perception of oxidation in beef. Meat Sci..

[105] Verma S.P., Sahoo J. (2000). Improvement in the quality of ground chevon during refrigerated storage by tocopherol acetate preblending. Meat Sci..

[106] Greene B.E., Cumuze T.H. (1982). Relationship between TBA numbers and inexperienced panelists’ assessments of oxidized flavor in cooked beef. J. Food Sci..

[107] Serra A., Conte G., Giovannetti M., Casarosa L., Agnolucci M., Ciucci F., Palla M., Bulleri E., Cappucci A., Servili M. (2018). Olive pomace in diet limits lipid peroxidation of sausages from cinta senese swine. Eur. J. Lipid Sci. Technol..

[108] Kotsampasi B., Bampidis V.A., Tsiaousi A., Christodoulou C., Petrotos K., Amvrosiadis I., Fragioudakis N., Christodoulou V. (2017). Effects of dietary partly destoned exhausted olive cake supplementation on performance, carcass characteristics and meat quality of growing lambs. Small Rumin. Res..

[109] Hamdi H., Majdoub-Mathlouthi L., Picard B., Listrat A., Durand D., Znaïdi I.A., Kraiem K. (2016). Carcass traits, contractile muscle properties and meat quality of grazing and feedlot Barbarine lamb receiving or not olive cake. Small Rumin. Res..

[110] Ozdogan M., Ustundag A.O., Yarali E. (2017). Effect of mixed feeds containing different levels of olive cake on fattening performance, carcass, meat quality and fatty acids of lambs. Trop. Anim. Health Prod..

[111] Sucu E., Akbay K.C., Şengül Ö., Yavuz M.T., Ibrahim A.K. (2018). Effects of stoned olive pomace on carcass characteristics and meat quality of lambs. Turkish J. Vet. Anim. Sci..

[112] Liotta L., Chiofalo V., Presti V. Lo, Chiofalo B. (2019). In vivo performances, carcass traits, and meat quality of pigs fed olive cake processing waste. Animals.

[113] Chiofalo V., Liotta L., Presti V. Lo, Gresta F., Di Rosa A.R., Chiofalo B. (2020). Effect of dietary olive cake supplementation on performance, carcass characteristics, and meat quality of beef cattle. Animals.

[114] Munekata P.E.S., Rocchetti G., Pateiro M., Lucini L., Domínguez R., Lorenzo J.M. (2020). Addition of plant extracts to meat and meat products to extend shelf-life and health-promoting attributes: An overview. Curr. Opin. Food Sci..

[115] Martínez L., Ros G., Nieto G. (2018). Hydroxytyrosol: Health benefits and use as functional ingredient in meat. Medicines.

[116] Lorenzo J.M., Domínguez R., Carballo J., Banerjee R., Verma A.K., Siddiqui M.W. (2017). Control of lipid oxidation in muscle food by active packaging technology. Natural Antioxidants: Applications in Foods of Animal Origin.

[117] Umaraw P., Munekata P.E.S., Verma A.K., Barba F.J., Singh V.P., Kumar P., Lorenzo J.M. (2020). Edible films/coating with tailored properties for active packaging of meat, fish and derived products. Trends Food Sci. Technol..

[118] Domínguez R., Barba F.J., Gómez B., Putnik P., Bursać Kovačević D., Pateiro M., Santos E.M., Lorenzo J.M. (2018). Active packaging films with natural antioxidants to be used in meat industry: A review. Food Res. Int..

[119] Djenane D., Gómez D., Yangüela J., Roncalés P., Ariño A. (2019). Olive leaves extract from algerian oleaster (*Olea europaea* var. Sylvestris) on microbiological safety and shelf-life stability of raw halal minced beef during display. Foods.

[120] Aouidi F., Okba A., Hamdi M. (2017). Valorization of functional properties of extract and powder of olive leaves in raw and cooked minced beef meat. J. Sci. Food Agric..

[121] Muíño I., Díaz M.T., Apeleo E., Pérez-Santaescolástica C., Rivas-Cañedo A., Pérez C., Cañeque V., Lauzurica S., de la Fuente J. (2017). Valorisation of an extract from olive oil waste as a natural antioxidant for reducing meat waste resulting from oxidative processes. J. Clean. Prod..

[122] Elama C., Tarawa M., Al-Rimawi F. (2017). Oleuropein from olive leaf extract as natural antioxidant of frozen hamburger. J. Food Sci. Eng..

[123] Hawashin M.D., Al-Juhaimi F., Ahmed I.A.M., Ghafoor K., Babiker E.E. (2016). Physicochemical, microbiological and sensory evaluation of beef patties incorporated with destoned olive cake powder. Meat Sci..

[124] Nieto G., Martínez L., Castillo J., Ros G. (2017). Hydroxytyrosol extracts, olive oil and walnuts as functional components in chicken sausages. J. Sci. Food Agric..

[125] Kurt Ş., Ceylan H.G. (2017). Effects of olive leaf extract on the oxidation stability and microbiological quality of dry fermented sausage (Sucuk). Carpathian J. Food Sci. Technol..

[126] Bermúdez-Oria A., Rodríguez-Gutiérrez G., Rubio-Senent F., Fernández-Prior Á., Fernández-Bolaños J. (2019). Effect of edible pectin-fish gelatin films containing the olive antioxidants hydroxytyrosol and 3,4-dihydroxyphenylglycol on beef meat during refrigerated storage. Meat Sci..

[127] Moudache M., Nerín C., Colon M., Zaidi F. (2017). Antioxidant effect of an innovative active plastic film containing olive leaves extract on fresh pork meat and its evaluation by Raman spectroscopy. Food Chem..

[128] Dua S., Bhat Z.F., Kumar S. (2015). Effect of oleuropein on the oxidative stability and storage quality of Tabaq-Maz, fried mutton ribs. Food Biosci..

